# Exploring the dietary strategies of phenylalanine: Improving muscle nutraceutical quality as well as muscle glycogen and protein deposition in adult grass carp (*Ctenopharyngodon idella*)

**DOI:** 10.1016/j.fochx.2024.101421

**Published:** 2024-04-27

**Authors:** Jing-Feng Han, Lin Feng, Wei-Dan Jiang, Pei Wu, Yang Liu, Ling Tang, Shu-Wei Li, Cheng-Bo Zhong, Xiao-Qiu Zhou

**Affiliations:** a Animal Nutrition Institute, Sichuan Agricultural University, Chengdu 611130, China; b Fish Nutrition and Safety Production University Key Laboratory of Sichuan Province, Sichuan Agricultural University, Chengdu 611130, China; c Key Laboratory of Animal Disease-Resistance Nutrition, Ministry of Education, Ministry of Agriculture and Rural Affairs, Key Laboratory of Sichuan Province, Sichuan 611130, China; d Animal Nutrition Institute, Sichuan Academy of Animal Science, Sichuan Animtech Feed Co. Ltd, Chengdu 610066, Sichuan, China

**Keywords:** Phenylalanine, Fillet quality, Amino acid, Fatty acid, Glucose metabolism, Protein deposition

## Abstract

Muscle is the main edible part of bony fish. The purpose of this study was to investigate the influences of phenylalanine (Phe) on muscle quality, amino acid composition, fatty acid composition, glucose metabolism, and protein deposition in adult grass carp. The diets at 2.30, 4.63, 7.51, 10.97, 13.53, and 17.07 g/kg Phe levels were fed for 9 weeks. The results manifested that Phe (10.97–13.53 g/kg) increased the pH of the fillets and decreased muscle cooking loss and lactic acid content; Phe (7.51–17.07 g/kg) improved the composition of the fillets in terms of flavor (free) amino acids, bound amino acids (especially EAA), and fatty acids (especially EPA and DHA); Phe (7.51–13.53 g/kg) increased muscle glycogen content (possibly related to the AMPK signaling pathway) and muscle protein deposition (possibly related to IGF-1/4EBP1/TOR and AKT/FOXOs signaling pathways). In conclusion, a diet with appropriate Phe levels could improve fillet quality.

## Highlights

• Phe improved muscle amino acid and fatty acid composition in grass carp.
• Phe enhanced muscle pH and glycogen content in grass carp.
• Phe promoted muscle protein synthesis; inhibited degradation in grass carp.
• Phe requirement was determined by PWG and muscle glycogen content.

## 1 Introduction

Over the last twenty years, fish has been crucial in global food production, and fish fillets have been acknowledged as essential for a healthy human diet due to their nutrient richness (Naylor et al., 2021). Not only are fillets a crucial source of animal protein supplement for human beings, it is also rich in nutrients such as polyunsaturated fatty acid (PUFA) and essential amino acid (EAA), among PUFAs, eicosapentaenoic acid (EPA) and docosahexaenoic acid (DHA) are especially notable for their important role in human health (Jiang et al., 2016). The nutrient content of the diet can influence fillet quality. For instance, proteins or amino acid compositions in diet might affect fillet quality (Dong et al., 2022; Wen et al., 2023). Phenylalanine (Phe), as one of the EAAs in animals, is not only a precursor substance for protein synthesis but also participates in glycolipid metabolism in the organism as one of the ketogenic amino acids (Guo et al., 2022). Previous studies have shown that an appropriate level of Phe could promote the growth of young grass carp (Li et al., 2015) and triploid rainbow trout (*Oncorhynchus mykiss*) (Zhang et al., 2023). Fish growth mainly involves the development of muscles (Xiao et al., 2023). Studies found muscle growth was closely linked to glucose metabolism (Liu et al., 2020) and protein deposition (Xiao et al., 2023). Studies on the effects of Phe on muscle glucose metabolism and protein deposition in fish are limited, and further research is necessary.

Glucose metabolism is divided into glycogen synthesis and decomposition. Glycogen synthesis is closely related to glucose transport, which is primarily dependent on glucose transporter 4 (GLUT4) (Lucia et al., 2012). Glycogenolysis is mainly a series of reactions in which key enzymes such as hexokinase (HK) and phosphofructokinase (PFK) decompose glycogen into pyruvate and lactic acid via the glycolysis pathway (Zhang et al., 2022). AMPK played an important role in glucose metabolism (Lucia et al., 2012), while Phe could up-regulate AMPK mRNA levels in carnivorous largemouth bass (*Micropterus salmoides*) (Yi et al., 2022). A previous study found herbivorous fish consume more carbohydrates than carnivorous fish (Zhou et al., 2016). Up to now, only one study reported the effects of Phe on fish glucose metabolism, with a focus on the mRNA levels of glycolytic enzymes and AMPK (Yi et al., 2022), and did not include comprehensive research on the specific mechanisms of glycogen synthesis and glycolysis. Phe could elevate blood insulin levels in mice (Zhou et al., 2022). Insulin could stimulate muscle glucose uptake by mobilizing GLUT4 (Jones et al., 2003), and increase the mRNA level of PFK in the muscle of grass carp (Zhang et al., 2024). These studies suggested a potential relationship between Phe and glucose metabolism, hence, which needs to be further investigated.

Fish muscle growth is closely linked to protein deposition (Jiang et al., 2021). Protein deposition mainly hinges on the equilibrium of protein synthesis and protein degradation (McCarthy & Esser, 2010). Protein synthesis is mainly regulated by the target of rapamycin (TOR)/ ribosomal protein S6 kinase 1 (S6K1)/eukaryotic translation initiation factor 4E (eIF4E) binding protein 1 (4EBP1) regulation of signaling pathway (Gao et al., 2019). However, the effects of Phe on the TOR signaling pathway have yielded conflicting results in different animal studies. A previous study found that Phe up-regulated TOR mRNA level in the gill of grass carp (Feng et al., 2017), but a study on human skeletal muscle showed that increased Phe content may reduce mTOR protein level (Trajkovic et al., 2014). The ubiquitin-proteasome system (UPS) can regulate protein degradation (Xiao et al., 2023). UPS is connected with phosphoinositide 3-kinase (PI3K)/AKT signaling pathway and forkhead box O (FOXO) protein transcription factors (von Haehling et al., 2017). No research has reported the influence of Phe on protein degradation in aquatic animals. Fish could convert Phe into tyrosine (Tyr) (Guo et al., 2022). Tyr phosphorylation can activate AKT signaling in cells (Chen et al., 2001). A study demonstrated that Phe can lower cysteamine (Cys) levels in triploid rainbow trout (*Oncorhynchus mykiss*) (Zhang et al., 2023). Cys could reduce the mRNA level of FOXO4 in the skeletal muscle of finishing pigs (Blachier et al., 2015). Given these intricate pathways and interactions, it is essential to conduct comprehensive and in-depth investigations into the effects of varying dietary Phe levels on muscle protein synthesis and degradation in fish.

At present, studies have shown that amino acids (tryptophan and threonine) can improve the muscle quality of grass carp, and both tryptophan and threonine increased the content of Phe in the muscle of grass carp (Wen et al., 2023; Xiao et al., 2023), but the effect of Phe on the quality of grass carp fillets has not been reported. Therefore, this study aimed to probe into the effects of dietary Phe on muscle amino acid and fatty acid composition, glucose metabolism, and protein deposition of grass carp. Additionally, this study aimed to assess the influences of Phe on the muscle quality of grass carp. At the same time, this study also identified the adult grass carp's Phe requirement, which perhaps offered a down-to-earth guide for enacting the commercial aquafeed and finally contributed to producing high-quality aquatic products for humans.

## 2 Materials and methods

### 2.1 Experimental diet preparation and feeding trial

Table S1 displays the experimental formula used in this study, consisting of fish meal, gelatin, corn starch, α-starch, and a crystal amino acid mixture as the primary components. Essential amino acids (EAAs) are supplemented to fulfill the amino acid requirements of grass carp, except for Phe. L-Phe was supplemented to the basal diet to produce six diets containing graded levels of Phe (0.00, 3.00, 6.00, 9.00, 12.00, and 15.00 g/kg). L-glycine was used to maintain dietary isonitrogen as described in a previous study (Li et al., 2015). All the elements were mixed, added the appropriate amount of water and extruded with a screw Extruder (F-75, Nanjing, China) to make a pellet mixture, the granules were air-seasoned and stockpiled at −20 °C for later use. The content of Phe in the test diet was determined by an L-8900 amino acid analyzer (Hitachi, Ltd., Tokyo, Japan), the measured concentrations of Phe in the six treatment diets were 2.30 (basal diet), 4.63, 7.51, 10.97, 13.53 and 17.07 g/kg diet.

Grass carp was obtained from a farm in Sichuan (China). Underwent a 4-week adaption period, 450 grass carp (average body weight 520.90 ± 1.98 g) were randomly placed in 18 cages (1.4 × 1.4 × 1.4 m), with 25 fish in each cage, each group included three replicates. The test fish was fed four times a day (7:00, 11:00, 15:00, and 19:00) for 9 weeks. During the trial period, dissolved oxygen was ≥6.0 mg/L, water temperature was 28.2 ± 4.2 °C, and pH was 7.8–8.1. The experiment was conducted outdoors under natural light.

### 2.2 Sample collection and analysis

After the test, the proprietary fish underwent fasting. Following the fasting period, the fish in each cage were weighed to determine growth performance indicators. Blood samples were then taken from the tail veins of six fish in each group. Another 9 fish from each group were narcotized with benzocaine (50 mg/L) and sacrificed by a blow to the head for subsequent sample collection (Wen et al., 2023). The method of obtaining muscle samples is based on a previous study (Jiang et al., 2021). The pH value and cooking loss of the left dorsal muscle were measured. Meanwhile, the opposite side flesh was refrigerated in liquid nitrogen and then stored at −80 °C for the following biochemical analysis. The proximate composition of muscle (moisture, crude protein, and crude fat) is analyzed by the method illustrated by the International, AOAC, 2005.

The hepatopancreas was only used to measure the liveness of glutamate-oxaloacetate transaminase (GOT) and glutamate-pyruvate transaminase (GPT). The activity of TH and PAH in muscle was tested based on instructions from the ELISA kit (Shanghai Enzyme-Linked, Shanghai, China). The plasma ammonia content (PAC) of the tail vein was assayed by the enzyme two-point method, reference to methods described in previous study (Wang et al., 2015). GOT and GPT liveness of hepatopancreas and muscle, as well as the activity levels of pyruvate kinase (PK), PFK, and HK in muscle, the contents of pyruvic acid, and lactate, were confirmed by respective kits (Nanjing Institute of Bioengineering, Nanjing, Jiangsu, China).

Percent weight gain (PWG), specific growth rate (SGR), feed intake (FI), feed efficiency (FE), and flesh rate were calculated according to the method of the previous study (Wen et al., 2023).

### 2.3 Morphometric analysis and immunofluorescence

The muscle sample was fixed in a 4% paraformaldehyde solution, and the muscle tissue of appropriate size was embedded in paraffin. It is cut into 3–5 μm laminas and dyed with periodic acid Schiff (PAS) staining method from Servicebio in Wuhan, China. Nikon TS100 optical microscope was used for observation and Image J software was used to confirm muscle glycogen.

According to the SABC kit, the embedded paraffin samples were sliced and put into the oven at 60 °C for 1–2 h, after rehydration, inactivation, and antigen repair for washing, the slices were closed with goat serum sealing solution (5% BSA) for 1 h at indoor temperature and then hatched with P-AMPK (1:100, HUABIO, SD0810) primary antibody at 4 °C overnight. Dropwise addition of fluorescent secondary antibody (1:1000, Beyotime, China) was incubated at room temperature and protected from light for 1 h, stained with DAPI, blocked, and photographed for analysis with an inverted fluorescence microscope. The cumulative optical density (IOD) was quantified using Image Pro Plus® 4.5.

### 2.4 Fatty acids and amino acids analysis

The muscle's fatty acid composition was analyzed using gas chromatography. The method involved taking 200 mg of the sample, which was then immersed in a chloroform-methanol mixture (2:1) for extraction. Subsequently, KOH-CH3OH was added for saponification, followed by the addition of BF3-CH3OH solution, which was mixed thoroughly. The mixture was then subjected to a 20 min incubation in a water bath at 85 °C. Finally, hexane and NaCl solution were added for extraction, and the fatty acid methyl esters were determined using a GC-2010 Plus instrument (Shimadzu, Kyoto, Japan).

The content of bound amino acids in muscle was determined by an automatic amino acid analyzer (L-8900). The method can be succinctly described as follows: infiltrate 100–200 mg of the sample with HCl solution, fill with nitrogen for 1 min, and close the hydrolysis tubes; hydrolyze the sample at high temperature for 22–24 h. Distilled water was fixed to 100 mL, and 200 μL of the filtered upper solution was taken, dried, and added to the HCl solution, and then measured after mixing. The free amino acid composition of muscle was analyzed by a full-automatic amino acid analyzer (L-A8080). In brief, muscle samples were homogenized, and 400 μL of muscle homogenate was mixed with 800 μL of salicylic acid standard solution for 30 min at indoor temperature, and then centrifuged and filtered to remove the supernatant for determination.

### 2.5 Quantitative real-time PCR analysis

RNA was extracted from muscle using RNAiso Plus Kit (Takara, Dalian, China), and RNA was inverse transcribed to cDNA according to the specification of PrimeScript™ RT reagent Kit (Takara, Dalian, China). Moreover, the RNA was amplified by fluorescence quantitative PCR (using qPCR and SYBR). Our laboratory previously conducted internal reference gene screening (data not shown) and selected β-actin as the reference gene. The mRNA levels of the desired genes were counted using the 2^-ΔΔCT^ method. Table S2 shows the primer sequences.

### 2.6 Western blotting

Muscle tissue was lysed to obtain protein samples, which were then separated on an SDS-PAGE gel and transferred to a PVDF membrane. After sealing, the PVDF membrane was placed in the primary antibody and incubated at 4 °C overnight; after that, the PVDF membrane was hatched with the secondary antibody for 1.5 h at room temperature. Color development was achieved with the ECL imaging kit (Affinity, China). Quantitative analysis was conducted using Image Lab 5.2 software.

### 2.7 Statistical analysis

Data statistics of this test were analyzed using one-way ANOVA and Duncan's multiple interval test in SPSS 22.0 software, *P* < 0.05 was a significant discrepancy, and data were shown as means ± standard deviation (SD). Based on the platform model of this experiment, the *P*-values and R^2^ of one-dimensional regression, quadratic regression, broken line regression, and triple regression were compared and analyzed, and the broken line regression model was selected to determine the Phe requirement of adult grass carp.

## 3 Results

### 3.1 Influences of dietary Phe on growth performance and amino acid metabolism in grass carp

Table 1 presents the influences of varying dietary Phe levels on the production performance of grass carp. FBW, PWG, SGR, FI, and FE were significant increases at the Phe level of 4.63 g/kg (*P* < 0.05), with no significant changes at 7.51–17.07 g/kg Phe levels (*P* > 0.05). In comparison to the Phe deficiency group, the meat content in the diet groups with Phe levels of 10.97 g/kg and 17.07 g/kg exhibited significant increases (*P* < 0.05). As the Phe level increased, the PAC level initially decreased before increasing, in the 10.97 g/kg Phe group reached the minimum; liver GOT and GPT levels increased, peaking at 13.53 g/kg and 10.97 g/kg Phe levels (*P* < 0.05), respectively, before gradually decreasing; muscle PAH and TH levels peaked in the 10.97 g/kg diet Phe group (*P* < 0.05), while the maximum levels of GOT and GPT were in the 17.07 g/kg diet Phe group (*P* < 0.05).

**Table 1:** Effects of dietary Phe on growth performance and amino acid metabolism of adult grass carp.

Item	Dietary Phe levels(g/kg diet)					
	2.30	4.63	7.51	10.97	13.53	17.07
IBW^1^	520.77 ± 0.00	520.6 ± 0.90	521.00 ± 1.37	520.79 ± 1.19	520.61 ± 0.68	521.60 ± 1.23
FBW^1^	897.07 ± 47.24^a^	1061.33 ± 58.49^b^	1145.33 ± 30.47^c^	1171.47 ± 26.84^c^	1172.14 ± 34.42^c^	1207.67 ± 47.34^c^
PWG^1^	72.26 ± 0.09^a^	103.86 ± 0.11^b^	119.82 ± 0.05^c^	124.93 ± 0.05^c^	125.15 ± 0.07^c^	131.34 ± 0.09^c^
SGR^1^	0.86 ± 0.08^a^	1.13 ± 0.08^b^	1.25 ± 0.04b^c^	1.29 ± 0.03^c^	1.29 ± 0.05^c^	1.33 ± 0.06^c^
FI^1^	1014.27 ± 25.29^a^	1219.87 ± 18.19^b^	1421.79 ± 37.97^c^	1406.50 ± 33.37^c^	1405.17 ± 26.05^c^	1392.95 ± 27.59^c^
FE^1^	37.12 ± 4.76^a^	44.22 ± 4.19^b^	43.94 ± 1.56^b^	46.37 ± 2.98^b^	46.36 ± 1.99^b^	49.27 ± 4.31^b^
Flesh rate^1^	66.64 ± 2.44^a^	69.30 ± 4.82^ab^	68.63 ± 2.64^ab^	69.71 ± 2.29^b^	69.40 ± 1.34^ab^	70.29 ± 1.54^b^
Plasma						
PAC^2^	329.04 ± 7.17^c^	322.23 ± 14.39^bc^	311.75 ± 14.44^b^	293.41 ± 12.83^a^	308.61 ± 11.72^b^	318.04 ± 13.00^bc^
Hepatopancreas						
GOT^2^	1.32 ± 0.13^a^	2.93 ± 0.27^b^	5.71 ± 0.56^d^	5.31 ± 0.53^d^	5.82 ± 0.57^d^	4.43 ± 0.36^c^
GPT^2^	7.10 ± 0.73^a^	9.46 ± 0.92^b^	9.93 ± 0.87^b^	12.92 ± 1.12^c^	10.19 ± 0.90^b^	9.89 ± 1.01^b^
Muscle						
PAH 2	1303.93 ± 100.89^a^	1367.89 ± 74.01^a^	1435.91 ± 134.50^ab^	1581.08 ± 126.25^c^	1556.86 ± 127.02^bc^	1535.14 ± 86.40^bc^
TH^2^	703.06 ± 53.67^a^	744.12 ± 76.35^ab^	758.21 ± 76.58^ab^	796.20 ± 54.58^b^	769.73 ± 73.38^ab^	764.58 ± 59.28^ab^
GOT^2^	14.69 ± 1.14^a^	15.17 ± 1.41^a^	16.10 ± 1.35^a^	19.28 ± 1.12^b^	20.04 ± 1.93^b^	20.78 ± 1.07^b^
GPT^2^	3.00 ± 0.27^a^	5.06 ± 0.49^b^	6.94 ± 0.76^c^	7.22 ± 0.54^c^	6.74 ± 0.73^c^	8.17 ± 0.28^d^

1 Values are mean ± SD, n for 3 replicate groups, 25 fish per replicate. Different superscripts in the same row indicate significant differences (*P* < 0.05). IBW (g/fish): initial body weight; FBW (g/fish): final body weight; PWG (%): percent weight gain; SGR (%/d): specific growth rate; FI (g/fish): feed intake; FE (%): feed efficiency; Flesh rate (%).

2 Values are mean ± SD, n for 3 replicate groups, 2 fish per replicate. Different superscripts in the same row indicate significant differences (*P* < 0.05). GOT (U/g prot): glutamate oxaloacetate transaminase; GPT (U/g prot): glutamate-pyruvate transaminase; PAH (U/g tissue): Phenylalanine hydroxylase; TH (U/g tissue): Tyrosine hydroxylase; PAC (μmol/L): plasma ammonia contents.

### 3.2 Influences of dietary Phe on nutritional ingredient and physicochemical properties in grass carp muscle

Table 2 displays the impacts of dietary Phe on the nutritional elements and physicochemical attributes of grass carp muscle. In comparison with the deficient group, moisture and cooking losses were significantly reduced at 7.51 g/kg and 10.97 g/kg Phe level (*P* < 0.05), respectively. Crude protein and crude fat contents aggrandized with the level of Phe, both of which increased significantly at 10.97 g/kg dietary Phe group (*P* < 0.05). The pH (pH_0h_ and pH_24h_) and lactic acid content reached the minimum at 7.51 g/kg and 13.53 g/kg dietary Phe group (*P* < 0.05), respectively.

**Table 2:** Effects of dietary Phe on nutritional ingredient and physical and chemical properties of adult grass carp.

Item	Dietary Phe levels(g/kg diet)					
	2.30	4.63	7.51	10.97	13.53	17.07
Moisture^1^	81.00 ± 1.71^b^	79.78 ± 0.71^b^	78.04 ± 1.34^a^	76.45 ± 1.47^a^	77.07 ± 1.42^a^	77.05 ± 1.23^a^
Crude protein^1^	16.74 ± 1.15^a^	18.37 ± 1.16^b^	19.36 ± 0.44^bc^	20.04 ± 0.97^c^	19.92 ± 0.96^c^	19.46 ± 0.88^bc^
Crude lipid^1^	2.50 ± 0.28^a^	2.73 ± 0.11^ab^	2.95 ± 0.13^b^	3.32 ± 0.19^c^	3.50 ± 0.24^c^	3.28 ± 0.29^c^
Cooking loss^2^	9.64 ± 0.23^b^	9.59 ± 0.72^b^	8.79 ± 0.86^b^	6.23 ± 0.63^a^	6.23 ± 0.43^a^	6.20 ± 1.11^a^
pH_0h_^2^	6.580.13^ab^	6.60 ± 0.15^ab^	6.54 ± 0.11^a^	6.70 ± 0.18^bc^	6.76 ± 0.11^c^	6.77 ± 0.17^c^
pH_24h_^2^	6.07 ± 0.11^ab^	6.17 ± 0.11^ab^	6.06 ± 0.06^a^	6.14 ± 0.13^ab^	6.19 ± 0.17^b^	6.16 ± 0.13^ab^
Lactate^1^	1.73 ± 0.08^d^	1.51 ± 0.03^c^	1.55 ± 0.05^c^	1.24 ± 0.09^b^	1.06 ± 0.05^a^	1.57 ± 0.12^c^

Lactate (mmol/g prot); Cooking loss (%); Moisture (%); Crude lipid (%); Crude protein (%).

1 Values are mean ± SD, n for 3 replicate groups, 2 fish per replicate. Different superscripts in the same row indicate significant differences (*P* < 0.05).

2 Values are mean ± SD, n for 3 replicate groups, 3 fish per replicate. Different superscripts in the same row indicate significant differences (*P* < 0.05).

### 3.3 Influences of dietary Phe on the composition of amino acids and fatty acids in muscle of grass carp

**Fig. 1:**
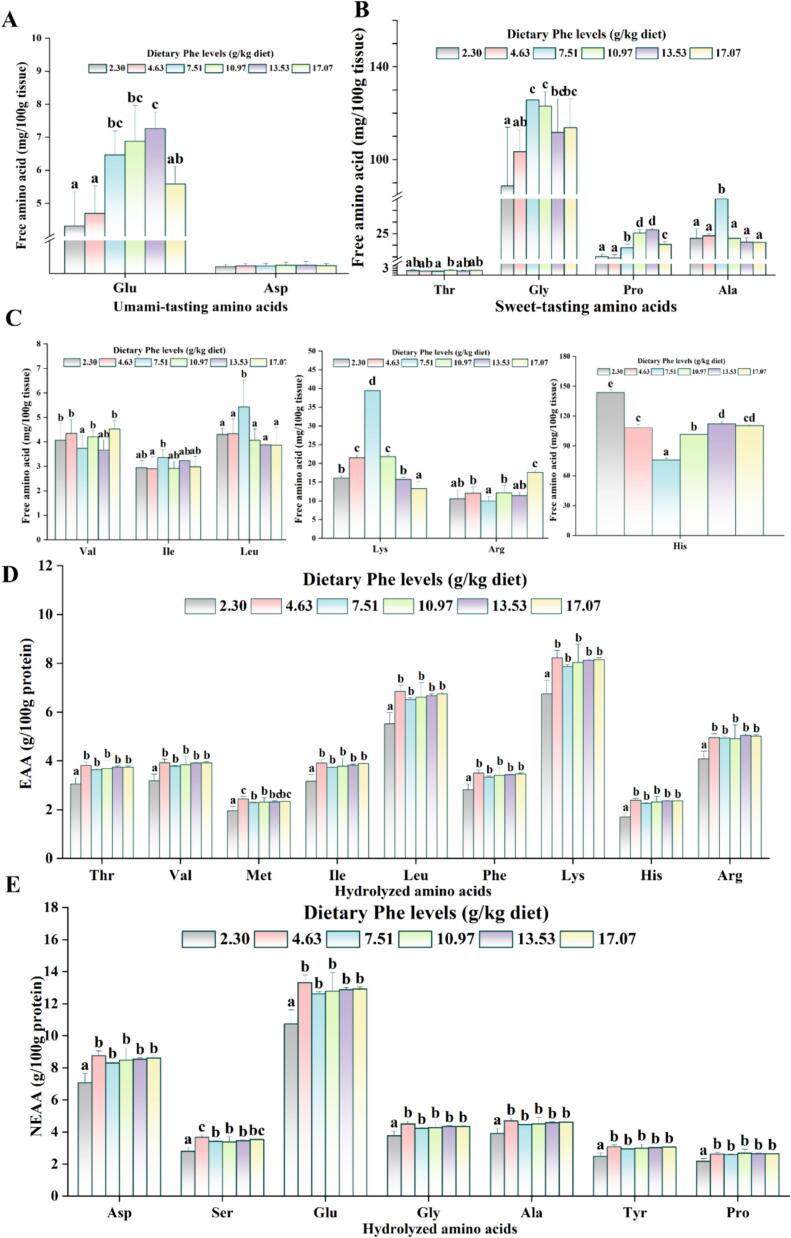
Effects of dietary Phe on the free amino acids (FAA) and bound amino acids composition in adult grass carp muscle. (A) Umami-tasting amino acids in muscle; (B) sweet-tasting amino acids in muscle; (C - E) other free amino acids in muscle; (F) EAA content of bound amino acids in muscle; (G) NEAA content of bound amino acids in muscle. Values are means ± SD, n for 3 replicate groups, 2 fish per replicate. Different letters indicate significant differences (*P* < 0.05).

The (A-E) within Fig. 1 depicts the varied effects of dietary Phe on the levels of free amino acids in grass carp muscle. As the dietary Phe level escalated, glutamic acid (Glu) content significantly increased at the 7.51–13.53 g/kg Phe level (*P* < 0.05), and the aspartate (Asp) content exhibited no significant alterations (*P* > 0.05). Compared with the Phe deficient group, threonine (Thr) and isoleucine (Ile) contents no significant difference in 4.63–17.07 g/kg Phe levels; glycine (Gly) and proline (Pro) contents significantly increased at 7.51–17.07 g/kg Phe level; alanine (Ala), leucine (Leu), and lysine (Lys) contents noticeably rose at the 7.51 g/kg Phe level (*P* < 0.05). With the increase of Phe level, the contents of valine (Val), arginine (Arg), and histidine (His) all showed a trend of first decreasing and then increasing, and all reached the minimum at 7.51 g/kg Phe level (*P* < 0.05). The influences of dietary Phe on the ingredient of bound amino acids of grass carp are shown in Fig. 1F-G. Compared to the lack group, at 4.63–17.07 g/kg Phe level, EAA (Thr, Val, methionine (Met), Ile, Leu, Phe, Lys, His and Arg) and NEAA (Asp, Ser, Glu, Gly, Ala, Tyr, and Pro) content was remarkably elevated (*P* < 0.05) and were no distinct difference at 7.51–17.07 g/kg Phe levels (*P* > 0.05).

**Fig. 2:**
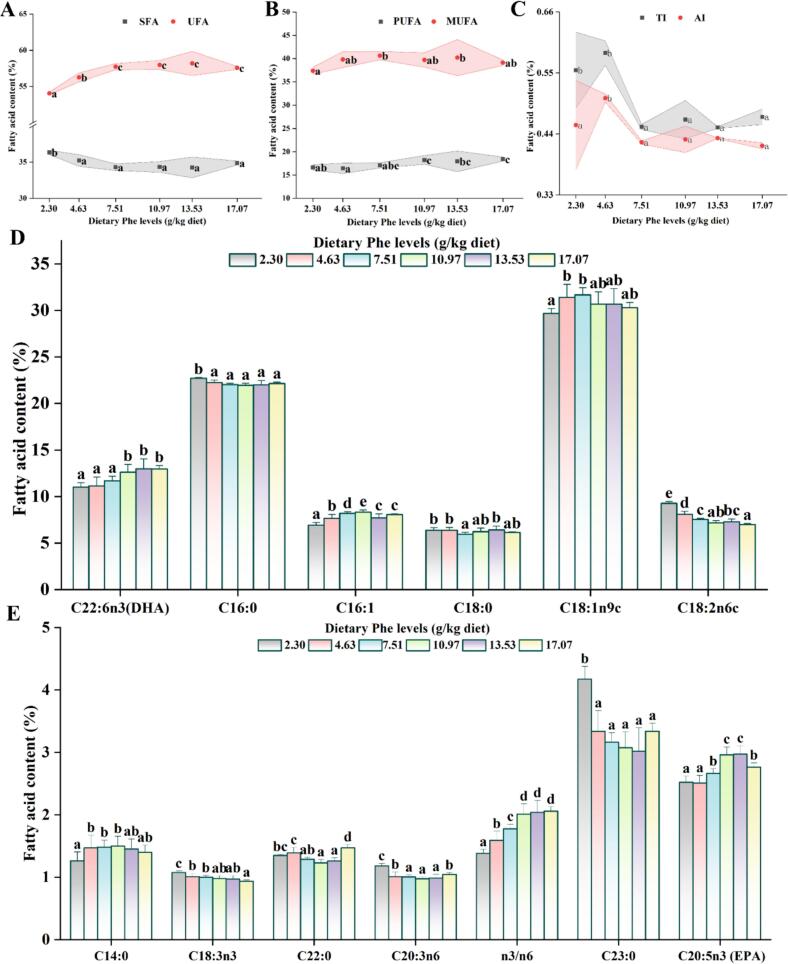
Effects of dietary Phe on the fatty acid (FA) profile (% of total FA methyl esters) in adult grass carp muscle. (A) Monounsaturated fatty acid (MUFA) and Polyunsaturated fatty acids (PUFA); (B) Saturated fatty acid (SFA) and Unsaturated fatty acid (UFA); (C) Atherogenicity index (AI) and Thrombogenicity index (TI); (D/E) Other fatty acid (FA). Values are means ± SD, n for 3 replicate groups, 2 fish per replicate. Different letters indicate significant differences (*P* < 0.05).

The influences of added Phe on the fatty acid composition of grass carp muscle are shown in Fig. 2. With the increase of Phe level, MUFA and PUFA showed a gradually increasing trend, and reached the maximum at 7.51 and 17.07 g/kg Phe levels (*P* < 0.05), respectively; C22:6n3 and C16:1 increased significantly at 10.97 g/kg Phe level (*P* < 0.05). Compared with the Phe deficient group, UFA, C14:0, and C18:1n9c were significantly increased and SFA was significantly decreased at 4.63 g/kg Phe level (*P* < 0.05); TI and AI were significantly decreased at 7.51 g/kg Phe level (*P* < 0.05); α-linolenic acid (C18:3n3), linoleic acid (C18:2n6c), C20:3n6, C23:0, and C16:0 markedly decreased at 4.63–17.07 g/kg Phe levels (*P* < 0.05).

### 3.4 Influences of Phe on relevant parameters to glucose metabolism

**Fig. 3:**
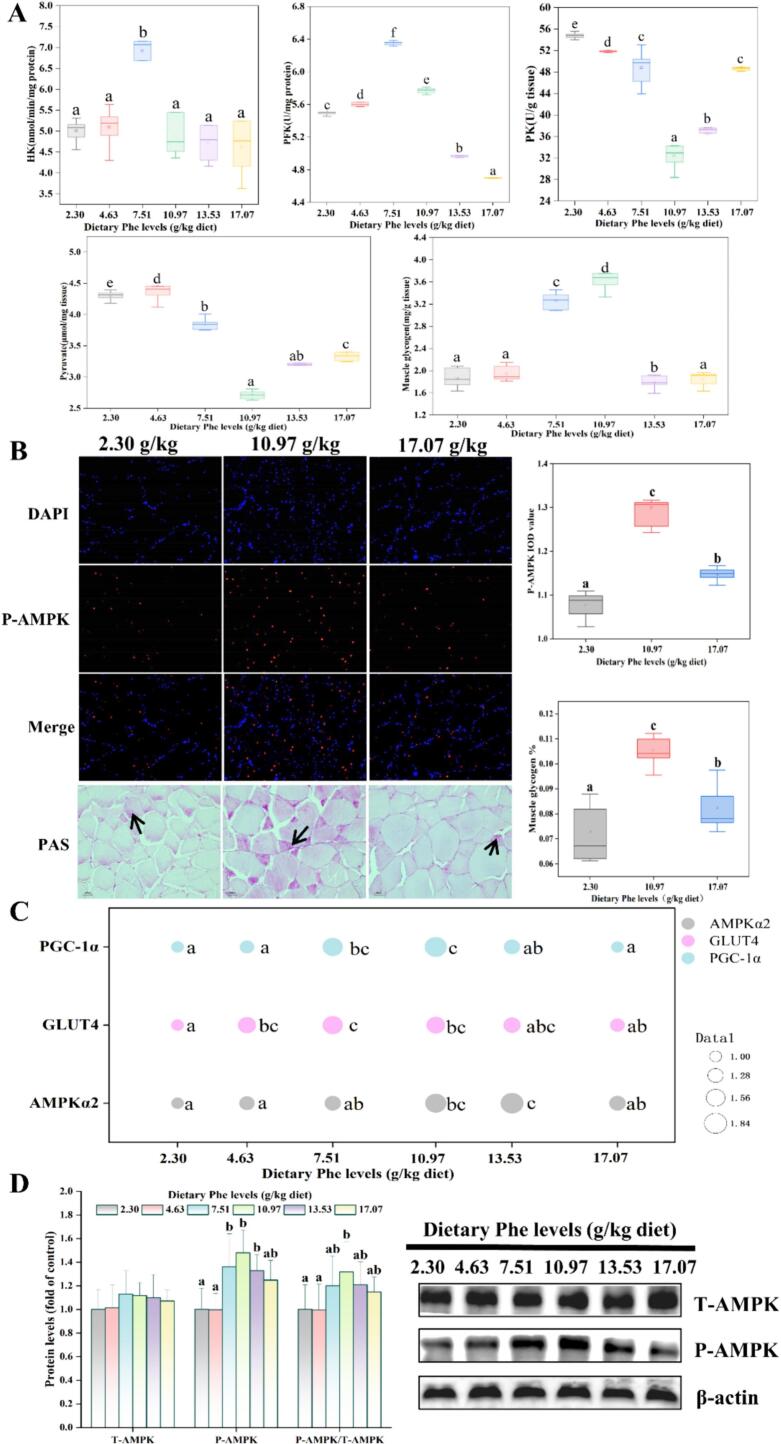
Effect of feed addition of Phe on glycogen content in muscle of grass carp. (A) Enzyme activities and content of glucose metabolism indexes, n for 3 replicate groups, 2 fish per replicate; (B) PAS staining and P-AMPK Immunofluorescence of muscle, purple part is glycogen (black arrows); (C) Bubble plot of mRNA levels associated with muscle glucose metabolism pathways, n for 3 replicate groups, 2 fish per replicate; (D) The protein levels of T-AMPK, P-AMPK, and P-AMPK/ T- AMPK, n for 3 replicate groups, 2 fish per replicate. Values are means ± SD. Mean values within the same row with different superscripts are significantly different (*P* < 0.05). HK: hexokinase (nmol/min/mg protein); PFK: phosphofructokinase (U/mg protein); PK: pyruvate kinase (U/g tissue). (For interpretation of the references to color in this figure legend, the reader is referred to the web version of this article.)

As shown in Fig. 3A, muscle HK and PFK activities were significantly greater than those of the deficient group and reached their maximum at the dietary Phe level of 7.51 g/kg (*P* < 0.05). Compared with the Phe deficient group, muscle PK activity, and pyruvate content reached a minimum at 10.97 g/kg dietary Phe group, while muscle glycogen content reached a maximum (*P* < 0.05).

Fig. 3B displays muscle PAS staining and muscle P-AMPK immunofluorescence. Compared with the Phe deficient group, the P-AMPK IOD value, muscle glycogen content, P-AMPK protein level, and P/T-AMPK ratio were significantly higher at 10.97 g/kg dietary Phe group (*P* < 0.05). As shown in Fig. 3C, PGC-1α, and GLUT4 mRNA levels all increased with increasing levels of Phe and reached their maximum values at 10.97 and 7.51 g/kg Phe levels, respectively (*P* < 0.05).

### 3.5 Influences of Phe on correlation parameters to protein deposition of muscle

**Fig. 4:**
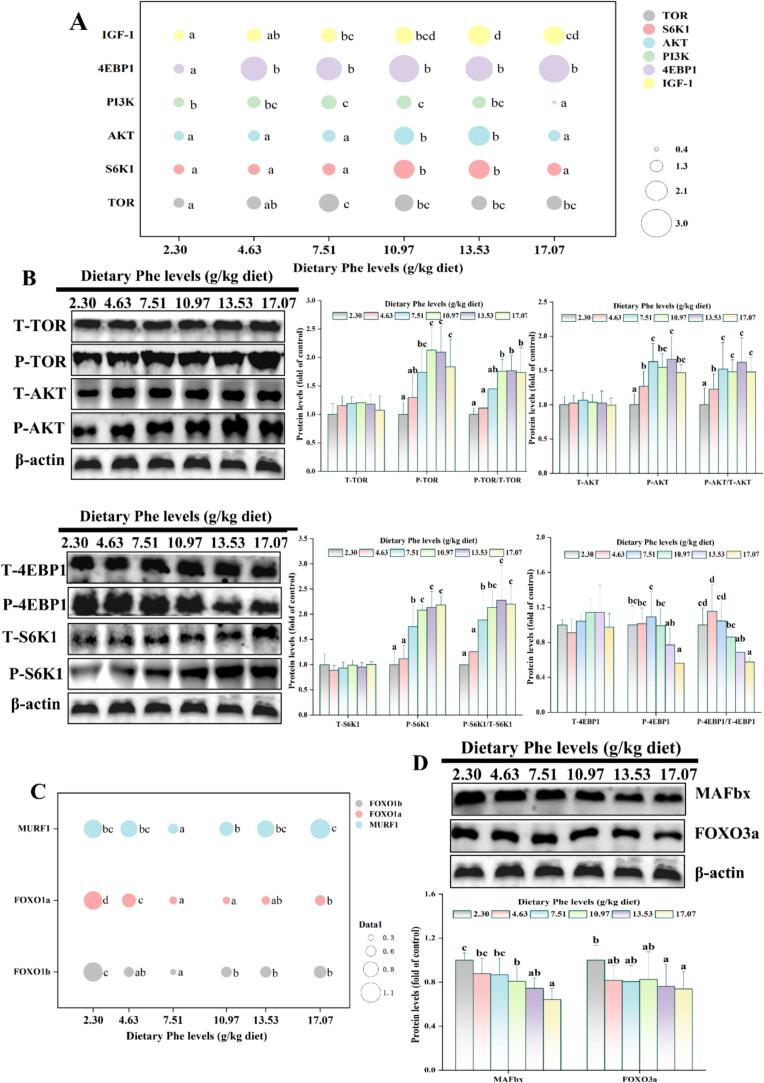
Influences of dietary Phe on parameters related to muscle protein synthesis and degradation in adult grass carp muscle. (A) Bubble plot of mRNA levels associated with muscle glucose metabolism pathways; (B) protein synthesis pathway-related protein levels in muscle; (C) Bubble plot of ubiquitin protease pathway-related mRNA levels in muscle; (D) Ubiquitin protease pathway-related protein levels in muscle. Values are means ± SD, n for 3 replicate groups, 2 fish per replicate. Different letters indicate significant differences (*P* < 0.05).

The relevant parameters to protein synthesis of muscle are shown in Fig. 4A-B. As Phe levels increased, the mRNA levels of IGF-1, 4EBP1, PI3K, AKT, S6K1, and TOR exhibited an initial rise followed by a gradual decline; reached the maximum at 13.53, 17.07, 10.97, 13.53, 13.53 and 7.51 g/kg Phe levels, respectively (*P* < 0.05); the protein levels of T-TOR, T-AKT, T-4EBP1, and T-S6K1 in muscle were remarkably unaffected (*P* > 0.05). Compared with the Phe deficient group, at 7.51 g/kg Phe level, the protein levels of P-TOR, P-AKT, P-S6K1, and P-4EBP1 were significantly higher, P/T-TOR and P/T-4EBP1 ratio in muscle were significantly increased at 10.97 and 13.53–17.07 g/kg Phe level, respectively; P/T-AKT and P/T-S6K1 ratio in muscle were significantly increased at 7.51 g/kg Phe level (*P* < 0.05).

Fig. 4C-D showed the effects of dietary Phe on parameters related to muscle protein degradation. The mRNA levels of MURF1, FOXO1a, and FOXO1b decreased initially, then increased as the Phe levels increased, and were all significantly lower at 7.51 g/kg Phe level (*P* < 0.05). Compared with the Phe deficiency group, MAFbx protein level significantly decreased at 10.97–17.07 g/kg Phe level, and FOXOx3a protein level significantly decreased at 13.53–17.07 g/kg Phe levels (*P* < 0.05).

## 4 Discussion

### 4.1 Dietary Phe improved the growth performance and amino acid metabolism in grass carp

In this study, appropriate Phe (7.78 g/kg diet) supplementation to the basal diet boosted the growth performance (PWG, FI, and FE) of adult grass carp. This aligns with findings from our previous research on young grass carp (Li et al., 2015). A previous study has suggested that enhanced fish growth could be linked to enhanced amino acid utilization (Dong et al., 2017). As reflected by decreased PAC levels and increased levels of GOT and GPT in both the hepatopancreas and muscle (Dong et al., 2017; Xiao et al., 2023). Our experimental results demonstrated that appropriate Phe supplementation led to a reduction in PAC levels and enhanced activities of GOT and GPT in the hepatopancreas and muscle. They are indicating that Phe may improve the availability of amino acids to promote fish growth. The metabolism of Phe is integral to its nutritional effects, with Phe being primarily converted into Tyr via PAH (Merrifield et al., 2013), subsequently metabolized into catecholamines under the action of TH (Guo et al., 2022), and finally plays a role in the body. Our findings indicated that Phe enhanced the activity of both PAH and TH. These results point towards a potential regulatory role of Phe metabolism via PAH and TH in mediating the nutritional benefits of Phe.

### 4.2 Dietary Phe improved flesh quality

The muscle, which makes up 60% of the fish body, is the main edible part of bony fish (Fuentes et al., 2010). In our study, we discovered that a suitable level of dietary Phe could improve the flesh rate. We next discussed the influences of dietary Phe supplementation on the muscle quality of grass carp.

The quality of fish muscle is closely tied to factors such as nutrition, health care, and physical-chemical characteristics, as highlighted in previous studies (Jiang et al., 2016; Xie et al., 2021). Key parameters including muscle protein and fat content, as well as the composition of amino acids and fatty acids, are crucial in determining the nutritional quality of fish muscle. The results showed that dietary Phe enhanced the content of muscle fat and protein in grass carp. Similar results were found in triploid rainbow trout (Zhang et al., 2023). Free amino acids (FAA) are considered taste-active compounds, participating in the Maillard reaction and imposing profound influences on the final flavor (Reina et al., 2014). Among these amino acids, Glu and Asp exert an umami taste, and Thr, Gly, Pro, and Ala exert a sweet taste (Hanagasaki & Asato, 2018). In the present study, we found that proper Phe level did not affect the content of Asp while augmenting the content of Glu, Thr, Gly, Pro, and Asp. The results manifested that the optimal level of Phe could boost the umami and sweetness of fish muscle, the enhancement of muscle umami taste primarily results from the elevated Glu content, as opposed to Asp. Bound amino acids are the main components of proteins (Xiao et al., 2023), and their compositions can be used as an index to judge their nutritional value. It was observed that Phe could increase the levels of bound amino acids in EAA (Thr, Val, Met, Ile, Leu, Phe, Lys, His, and Arg) and NEAA (Asp, Ser, Glu, Gly, Ala, Tyr, and Pro), suggesting that dietary Phe has the potential to enhance the nutritional value of muscle by positively influencing the composition of bound amino acids within the muscle.

Fish muscle provides a rich composition of fatty acids, especially EPA and DHA, which help to prevent cardiovascular diseases, while saturated fatty acids (SFA) increase the incidence of cardiovascular diseases (Jiang et al., 2016). The results of the present study revealed that appropriate levels of Phe enhanced the content of EPA, DHA, and total PUFA content as well as decreased SFA content. Furthermore, the health benefits of fatty acids can also be assessed by the atherosclerosis index (AI) and thrombogenicity index (TI) (Chen & Liu, 2020), which indicate the ratio between SFA and UFA, and the ratio between anti-thrombotic fatty acids and pro-thrombotic fatty acids, respectively. In the results of the present study, appropriate levels of Phe were found to reduce AI and TI in fish fillets. Therefore, the above results suggest that appropriate levels of Phe may enhance the health benefits of fish fillets in humans.

Physical-chemical qualities (cooking loss and pH) are also significant aspects of muscle mass. The rise in water holding capacity (WHC) of fillets results from decreased cooking losses (Jiang et al., 2016). This study found that adding Phe to the ration appropriately decreased cooking losses. Research has demonstrated that optimal Phe levels can enhance the WHC of fish fillets. Furthermore, muscle glycogen level significantly influences the postmortem muscle pH change by being converted to lactic acid through glycolysis (Silva et al., 2012). In this study, it was found that the appropriate addition of Phe increased muscle pH value and reduced lactic acid content. This may be linked to muscle glucose metabolism, leading us to investigate this further in our next study.

### 4.3 Dietary Phe improves muscle glucose metabolism in fish

Glucose metabolism primarily involves glycogen uptake and utilization. In our study, dietary Phe increases muscle glycogen content, which may be related to glucose uptake. GLUT4 plays a vital role in stimulating glucose uptake in fish skeletal muscle (Lucia et al., 2012). It has been found in myocytes that peroxisome proliferator-activated receptor γ coactivator 1α (PGC-1α) can positively regulate GLUT4 (Liu et al., 2020). In this study, dietary Phe augmented the mRNA levels of GLUT4 and PGC-1α, suggesting that Phe up-regulation of GLUT4 may be related to its up-regulation of PGC-1α. PGC-1α up-regulation may be associated with AMPK. AMPK could up-regulate PGC-1α in the muscle of olive flounder (*Paralichthys olivaceus*) (Liu et al., 2020). The current study showed that dietary Phe increased the levels of AMPK mRNA and phosphorylation. Further analysis showed a comparable trend in the levels of phosphorylated AMPK and PGC-1α mRNA. These results indicate that Phe may enhance AMPK/PGC-1α/GLUT4 signaling to boost muscle glucose uptake.

Glycolysis is the main process for utilizing glycogen in the endomysium. Muscle glycogen can be converted to pyruvate via the glycolysis pathway (Wang et al., 2021). Our findings showed that dietary Phe increased muscle glycogen levels and reduced muscle pyruvate content. This outcome could be attributed to the Phe effect on enzymes linked to glycogen metabolism during glycolysis. The conversion of glycogen to pyruvate by glycolysis is regulated by HK, PFK, and PK enzymes (Wang et al., 2021). Our findings indicated that dietary Phe elevated the activity of HK and PFK while reducing the activity of PK. Hence, we theorize that Phe might hinder the conversion of glycogen to pyruvate, leading to higher glycogen levels and lower pyruvate levels.

It is interesting to note that dietary Phe enhanced the activity of muscle PFK in grass carp, but decreased the activity of hepatic PFK in juvenile swimming crabs (Guo et al., 2022), which is potentially due to tissue-specific expression of PFK. It has been suggested that the expression patterns of PFK family genes vary across different tissues of grass carp (Zhang et al., 2024). The studies mentioned above support our conjecture, but further researches are required to verify this.

### 4.4 Dietary Phe increased muscle protein deposition in fish

Muscle protein deposition is also an important factor in muscle quality, which is determined by the equilibrium between protein synthesis and degradation (Jiang et al., 2021; Xiao et al., 2023). Previous studies have demonstrated that the TOR/S6K1/4EBP1 signaling pathway can positively regulate mammalian protein synthesis (Schiaffino et al., 2013). In our study, we found that the dietary addition of Phe augmented phosphorylation and mRNA levels of TOR/S6K1 and 4EBP1 in grass carp muscle. These findings indicate that Phe may enhance muscle protein synthesis in grass carp. Activation of the TOR pathway by Phe might be associated with the PI3K/AKT pathway. P-AKT triggers the TOR signaling pathway, initiating protein translation and synthesis (Jiang et al., 2021). The results of our test found that dietary Phe augmented the mRNA levels of PI3K and AKT as well as increased the protein level of P-AKT. The correlation analysis demonstrated a positive correlation between phosphorylated TOR and AKT, which confirms our hypothesis. The effect of Phe on AKT may be related to IGF-1. IGF-1 activates the PI3K/AKT signaling pathway in auricular chondrocytes (Chen et al., 2018). In our study, we observed that dietary Phe augmented the mRNA level of IGF-1 in muscle. Correlation analysis found a positive relationship between P-AKT level and IGF-1 mRNA level. In summary, dietary Phe boosts protein synthesis in fish, likely through the activation of the IGF-1/4EBP1/TOR pathway.

UPS is one of the main ways of protein degradation (Schiaffino et al., 2013). Decreased levels of MAFbx and MURF1 signaling factors impede protein degradation (McCarthy & Esser, 2010). Our research results showed that consuming dietary Phe lessened the protein level of MAFbx and the mRNA level of MURF1. This could be connected to the FOXO transcription factor. FOXO protein could activate the MuRF1 gene in the skeletal muscle of mice (Waddell et al., 2008). In our study, we discovered that dietary Phe reduced the mRNA levels of FOXO1a and FOXO1b, as well as the protein level of FOXO3a. The correlation analysis showed a positive relationship between MAFbx and FOXO3a protein levels, additionally, the mRNA level of MURF1 and the protein level of MAFbx displayed a similar trend with both FOXO1a and FOXO1b mRNA levels, as well as FOXO3a protein levels. The regulatory effect of Phe on the signaling factors above may be related to AKT. AKT can negatively regulate FOXO transcription factors (Schiaffino et al., 2013). In this study, dietary Phe increased AKT mRNA and protein levels. Additional analysis revealed a negative correlation between FOXO3a protein levels and FOXO1a mRNA levels as well as phosphorylated AKT levels, while the mRNA level of FOXO1b showed an opposite trend to that of phosphorylated AKT levels. These findings suggest that dietary Phe may inhibit protein degradation through the regulation of MAFbx, MURF1, and FOXO signaling factor-mediated UPS.

### 4.5 Interesting finding: High levels of dietary Phe showed different production indicators than young grass carp

The study results showed that high dietary Phe levels did not affect the growth performance of adult grass carp, including PWG, FI, and FE. This differs from the results of previous studies in our laboratory. Previous studies in our laboratory have shown that high levels of dietary Phe can negatively impact the germination of young grass carp (Li et al., 2015). This might be correlated with the different growth phases of grass carp. A study found that high dietary protein levels can hinder the growth of young grass carp (Xu et al., 2016), but do not affect sub-adult grass carp (Dong et al., 2022). The aforementioned research confirms our hypothesis; however, more investigation is necessary. The above research supports our hypothesis, but further study is needed.

### 4.6 Dietary Phe requirements of adult grass carp

In our study, the adult grass carp's dietary Phe requirement was found to be 7.78 g/kg diet (29.75 g/kg protein), which was lower than that of young grass carp 10.40 g/kg diet (34.40 g/kg protein), based on the broken-line regression analysis on the PWG (Li et al., 2015). This might be attributed to the faster growth rate of young grass carp compared to adult grass carp. Based on the crude protein broken-line regression analysis, the optimal dietary Phe requirement for adult grass carp was determined to be 10.47 g/kg diet (40.04 g/kg protein). The ideal Phe addition based on crude protein content exceeded the ideal Phe addition based on growth performance. This may be related to the activity of organisms. Fish spend most of their time in motion, and organismal activity increases the absorption of nutrients such as protein by muscle tissue (Yu et al., 2015). Therefore, the fish may need to increase muscle glycogen to sustain its motor capacity. Nevertheless, the specific causes require further investigation.

## 5 Conclusion

In this study, the effects of dietary Phe replenishment on growth performance, muscle quality, glucose metabolism, and protein deposition in adult grass carp were investigated. Three noteworthy findings emerged: (1) Adding optimal dietary Phe enhanced the nutrient content (crude protein and crude fat, among others), as well as the flavor-contributing amino acids (fresh and sweet) and nutraceutical fatty acids (EPA and DHA) in the muscle of adult grass carp. (2) Adding an optimal level of dietary Phe speeds up glucose uptake in grass carp muscle, leading to higher muscle glycogen content, possibly through the AMPK signaling pathway. (3) Optimal dietary Phe levels may enhance protein deposition by activating the TOR signaling pathway to promote muscle protein synthesis and by inhibiting protein degradation through negative regulation of the FOXO transcription factor. These results provide a theoretical basis and molecular mechanism for enhancing muscle quality by adding Phe to the ratio. At last, based on the broken-line regression analysis of PWG and crude protein, the dietary Phe requirements of adult grass carp were estimated as 7.78 g/kg diet (29.75 g/kg protein) and 10.47 g/kg diet (40.04 g/kg protein) (Fig. S1A).

The following are the supplementary data related to this article.

Supplementary material : Table S1**:** The components and nutritional makeup of the basal diet; **Table S2:** The real-time PCR primer sequences; **Table S3:** Correlation analysis of parameters in adult grass carp (*Ctenopharyngodon idella*) muscle; **Fig. S1:** Optimal Phe supplementation determined by broken-line regression analysis of PWG and muscle crude protein content of adult grass carp. Supplementary material

## Ethical statement

All experimental plans for this experiment were approved by the Animal Care Advisory Committee of Sichuan Agricultural University.

## CRediT authorship contribution statement

**Jing-Feng Han:** Visualization, Validation, Software, Investigation, Formal analysis, Data curation. **Lin Feng:** Supervision, Resources, Funding acquisition. **Wei-Dan Jiang:** Writing – review & editing, Supervision, Methodology, Investigation. **Pei Wu:** Validation, Methodology. **Yang Liu:** Methodology. **Ling Tang:** Resources. **Shu-Wei Li:** Resources. **Cheng-Bo Zhong:** Resources. **Xiao-Qiu Zhou:** Writing – review & editing, Supervision, Resources, Funding acquisition.

## Declaration of competing interest

The authors declare that they have no known competing financial interests or personal relationships that could have appeared to influence the work reported in this paper.

## Data Availability

The data that has been used is confidential.
