# Supplementary material for: Exploring the dietary strategies of phenylalanine: Improving muscle nutraceutical quality as well as muscle glycogen and protein deposition in adult grass carp (Ctenopharyngodon idella)
Source: Food Chem X. 2024 Apr 27;22:101421. doi: 10.1016/j.fochx.2024.101421 (PMC11096706; doi:10.1016/j.fochx.2024.101421)
Supplement: Supplementary material — : Table S1: The components and nutritional makeup of the basal diet; Table S2: The real-time PCR primer sequences; Table S3: Correlation analysis of parameters in adult grass carp (Ctenopharyngodon idella) muscle; Fig. S1: Optimal Phe supplementation determined by broken-line regression analysis of PWG and muscle crude protein content of adult grass carp. [file mmc1.docx]

**Supplementary materials**

**Exploring the dietary strategies of phenylalanine: improving muscle nutraceutical quality as well as muscle glycogen and protein deposition in adult grass carp (*Ctenopharyngodon idella*)**

Jing-Feng Han ^a^, Lin Feng ^a, b, c^, Wei-Dan Jiang ^a, b, c^, Pei Wu ^a, b, c^, Yang Liu ^a, b, c^, Ling Tang ^d^, Shu-Wei Li ^d^, Cheng-Bo Zhong ^d^, Xiao-Qiu Zhou ^a, b, c, *^

^a^ Animal Nutrition Institute, Sichuan Agricultural University, Chengdu, 611130, China

^b^ Fish Nutrition and Safety Production University Key Laboratory of Sichuan Province, Sichuan Agricultural University, Chengdu, 611130, China

^c^ Key Laboratory of Animal Disease-Resistance Nutrition, Ministry of Education, Ministry of Agriculture and Rural Affairs, Key Laboratory of Sichuan Province, Sichuan, 611130, China

^d^ Animal Nutrition Institute, Sichuan Academy of Animal Science, Sichuan Animtech Feed Co. Ltd, Chengdu, 610066, Sichuan, China

^*^ E-mail addresses: [*zhouxq@sicau.edu.cn*](mailto:zhouxq@sicau.edu.cn) *(X.-Q. Zhou).*

**Table S1**

The components and nutritional makeup of the basal diet.

| Ingredients | Content（%） | Ingredients^1^ | Content（%） |
| --- | --- | --- | --- |
| Fish meal | 3.00 | Crude protein^1^ | 26.15 |
| Gelatin | 8.50 | Crud fat^1^ | 4.54 |
| Amino acid mixture^2^ | 15.626 | Available phosphorus^1^ | 0.40 |
| Phe premix^3^ | 5.00 | n3^1^ | 1.02 |
| Corn starch | 22.079 | n6^1^ | 0.94 |
| α-starch | 29.00 |  |  |
| Fish oil | 2.62 |  |  |
| Soybean oil | 1.76 |  |  |
| Calcium dihydrogen phosphate | 1.90 |  |  |
| Vitamin premix^4^ | 1.00 |  |  |
| Mineral premixes^5^ | 2.00 |  |  |
| Choline chloride premix^6^ | 1.00 |  |  |
| Butylated hydroxyanisole (99%) | 0.015 |  |  |
| Microcrystalline cellulose | 6.50 |  |  |
| Total | 100.00 |  |  |

^1^ Values of crude protein and crude lipid content were measured. Available phosphorus, n-3 and n-6 contents were calculated values.

^2^ per kg amino acid mixture (g/kg): arginine, 3.42 g; histidine, 5.10 g; isoleucine, 8.39 g; leucine, 7.31 g; lysine, 10.19 g; cysteine, 1.11 g; tryptophan 3.09 g; tyrosine, 8.51 g; threonine, 8.45 g; valine, 7.28 g; methionine, 2.64 g, respectively.

^3^ per kg Phe premix, and the amount of glycine and microcrystalline cellulose was reduced to compensate. Per kilogram of Phe-glycine premix composition from diet 1 to 6 was as follows (g/kg): L- Phe (99.6%) 0.00, 60.24, 120.28, 180.72, 240.96, 301.20 g; glycine (99.5%) 137.02, 109.62, 82.21, 54.81, 27.40, 0.00 g and microcrystalline cellulose 868.98, 803.14, 797.31, 764.47, 731.63, 698.80 g, respectively.

^4^ Each kg of vitamin premix contains: Vitamin A acetate (500,000 IU/g), 0.39 g; VD_3_ (500,000 IU/g), 0.20 g; Vitamin E (50%), 23.23 g; VK_3_ (50%), 0.38 g; VB_12_ (1%), 0.94 g; D-Biotin (2%), 1.05 g; folic acid (95%), 0.17 g; Thiamine nitrate (98%), 0.11 g; VC (95%), 9.77 g; Niacin (99%), 3.44 g; Inositol (98%), 28.53 g; D-calcium pantothenate (98%), 4.20 g; Riboflavin (80%), 0.73 g; Vitamin B6 (98%), 0.45 g. All ingredients were diluted with corn starch to 1 kg.

^5^ Mineral premix per kg contains: MnSO_4_·H_2_O (31.8% Mn), 2.66 g; MgSO_4_·H_2_O (15.0% Mg), 256.79 g; FeSO_4_·H_2_O (30.0% Fe), 12.61 g; ZnSO_4_·H_2_O (34.5% Zn), 8.87 g; CuSO_4_·5H_2_O (25.1% Cu), 0.95 g; Ca (IO_3_)_2_ (3.2% I), 1.56 g; yeast selenium (0.2%), 13.65 g; All ingredients were diluted with corn starch to 1 kg.

^6^ Each kg choline chloride premix contains 261.89 g of choline chloride (50%). All ingredients were diluted with corn starch to 1 kg.

**Table S2**

The real-time PCR primer sequences as shown in Table S2.

| Target gene | Primer sequence forward (5′ → 3′) | Primer sequence reverse (5′ → 3′) | Accession number |
| --- | --- | --- | --- |
| *IGF-1* | GCTGCAGTTTGTGTGTGGAG | ATGCGATAGTTTCTGCCCCC | OL438919 |
| *PI3K* | AGTCAGTGCCTGTGGCTGAG | CGTGTCCATGACCTCAGAGC | KY763989 |
| *Akt* | CCTGGTGATGAAGGAGCTGA | CTGTCAGAGAGCCTCCAGCA | KY763985 |
| *TOR* | TCCCACTTTCCACCAACT | ACACCTCCACCTTCTCCA | JX854449 |
| *S6K1* | TGGAGGAGGTAATGGACG | ACATAAAGCAGCCTGACG | EF373673 |
| *4EBP1* | GCTGGCTGAGTTTGTGGTTG | CGAGTCGTGCTAAAAAGGGTC | KT757305 |
| *FOXO1a* | GCATCTCATAGCCATGCCCT | CACCTCCAAGATGACCGGAG | KP325483 |
| *FOXO1b* | CTCAACCTCATCTCGCCCAA | TCGGTATGGCGATTGGACTG | KP325484 |
| *MURF1* | TGTCTATGGACTACAGAGGAA | GGATTTCAAAGGAGGTTCAAG | JAIKLG010000006.1 |
| *MAFbx* | CGGACGAGATCTGGTTAGCC | GCTTGCGGATCTGTCTGTCT | (Zhou, et al., 2023) |
| *Glut4* | CCGTGCAGCCTACAGTTTTT | AGGAGAAAACCAGTGCAGCG | JAIKLG010000021.1 |
| *AMPKα2* | AGTTCTACCTGGCCTCCAGT | GAGAGCATCCAGAGGGCATC | MK294333.1 |
| *PGC-1α* | AAAGCCAGGGAAGCCAAGAG | ATGATGGGGAGGCAGAGGAT | JN195739 |
| *PYG* | TGCGGCATCTGGAAAGTTCT | TCCAGAGCTTCACGTGGTTC | JQ782458.1 |
| *GYS* | CCTCCAGTAACAACTCACAACA | CAGATAGATTGGTGGTTACGC | JQ792167.1 |
| *β-actin* | GGCTGTGCTGTCCCTGTA | GGGCATAACCCTCGTAGAT | M25013 |

**Table S3**

Correlation analysis of parameters in adult grass carp (*Ctenopharyngodon idella*) muscle.

| Independent parameters | Dependent parameters | Correlation coefficients | P-value |
| --- | --- | --- | --- |
| PGC-1α mRNA level | GLUT4 mRNA level | +0.807 | 0.052 |
| p-AMPK protein level | PGC-1α mRNA level | +0.799 | 0.057 |
| p-AKT protein level | p-TOR protein level | +0.916 | 0.010 |
|  | FOXO3a protein level | -0.825 | 0.043 |
|  | FOXO1a mRNA level | -0.976 | 0.001 |
|  | FOXO1b mRNA level | -0.786 | 0.064 |
| IGF-1 mRNA level | p-AKT protein level | +0.840 | 0.037 |
| FOXO3a protein level | MAFbx protein level | +0.887 | 0.019 |
|  | MURF1 mRNA level | +0.088 | 0.869 |
| FOXO1a mRNA level | MAFbx protein level | +0.624 | 0.185 |
|  | MURF1 mRNA level | +0.558 | 0.250 |
| FOXO1b mRNA level | MAFbx protein level | +0.329 | 0.524 |
|  | MURF1 mRNA level | +0.733 | 0.098 |

**Fig. S1.** Optimal Phe supplementation determined by broken-line regression analysis of PWG (A) and muscle crude protein content (B) of adult grass carp.

Zhou, Y., Wu, P., Jiang, W.-D., Liu, Y., Peng, Y., Kuang, S.-Y., Tang, L., Li, S.-W., Feng, L., & Zhou, X.-Q. (2023). Dietary cinnamaldehyde improves muscle protein content by promoting muscle fiber growth via PTP1B/IGF1/PI3K/AKTs-TOR/FOXO3a signaling pathway in grass carp (*Ctenopharyngodon idella*). *Food Chemistry, 399*. <https://doi,org/10.1016/j.foodchem.2022.133799>.

**Reference**
